# Une localisation rare au sphénoïde d’un kyste osseux anévrysmal

**DOI:** 10.11604/pamj.2017.27.231.12154

**Published:** 2017-07-31

**Authors:** Farida Abdoulkader, Kaoutar Aammou, Abdellatif Siwane, Fatiha Essodegui

**Affiliations:** 1 Service Central de Radiologie, Chu Ibn Rochd, Casablanca, Maroc

**Keywords:** Kyste osseux, anévrysmal, base du crâne, os sphénoïde, imagerie par résonance magnétique, Bone cyst, aneurysmal, base of the skull, sphenoid bone, magnetic resonance imaging

## Abstract

Le kyste osseux anévrysmal est une tumeur bénigne pouvant toucher tous les os du corps. Rare au niveau de la base du crane, il siège habituellement au niveau des os longs. Nous rapportons l'observation clinique d'un homme de 44 ans suivi depuis 9 mois pour une masse du basisphenoide étiquetée à tort comme un macroadénome invasif à prolactine et pour lequel il fut mis sous traitement (agonistes dopaminergiques). Nous avons redressé le diagnostic en évoquant le kyste osseux anévrysmal du sphénoïde de par son aspect radiologique en IRM quasi pathognomonique.

## Abstract (en)

Aneurysmal bone cyst is a benign tumor that can affect all bones in the body. It rarely affects the base of the skull. It is usually located at the level of the long bones. We report the clinical case of a 44-year old man treated for a mass in the basisphenoid wrongly diagnosed as invasive prolactin-producing macroadenoma for which he was put under dopamine agonists over a period of 9 months. We adjusted the diagnosis by suggesting aneurysmal bone cyst in the sphenoid bone on the basis of its almost pathognomonic radiological aspect on MRI.

## Introduction

Les kystes osseux anévrysmaux (KOA) sont des tumeurs osseuses bénignes qui surviennent en général chez les sujets jeunes âgés de moins de 30 ans [1]. Ils sont plus souvent localisés au niveau des os longs. L'atteinte de la base du crane et notamment du sphénoïde est inhabituelle [1, 2]. Il s'agit d'une lésion expansive faite de multiples logettes kystiques et hémorragiques. C'est avant tout une lésion bénigne qui ne nécessite pas systématiquement de recours à une chirurgie. Le diagnostic différentiel se pose fréquemment avec une autre lésion du basisphenoide, le macroadénome invasif sellaire. A travers une observation didactique, nous verrons comment se présente cette lésion en imagerie ainsi que les principaux diagnostics différentiels qu'elle pose dans ce siège.

## Patient et observation

**Figure 1:**
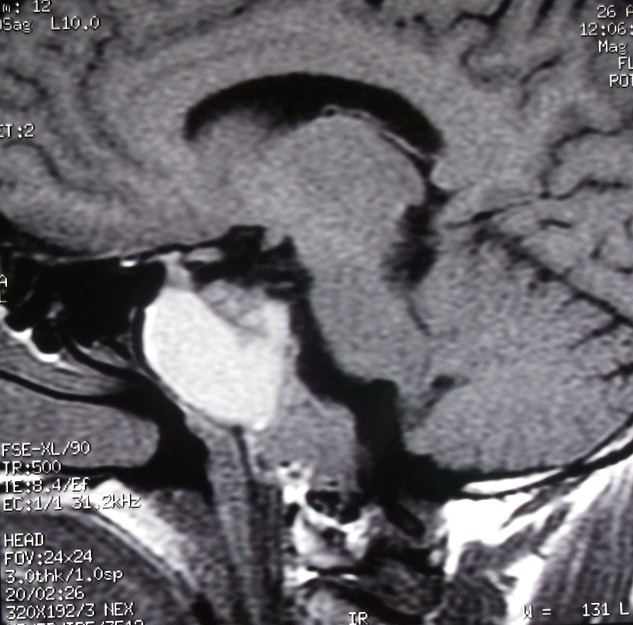
IRM coupe sagittale pondérée en T1 montrant un processus lésionnel solido-kystique centré sur le corps du sphénoïde fait de multiples logettes kystiques en hypersignal spontané avec niveaux

**Figure 2:**
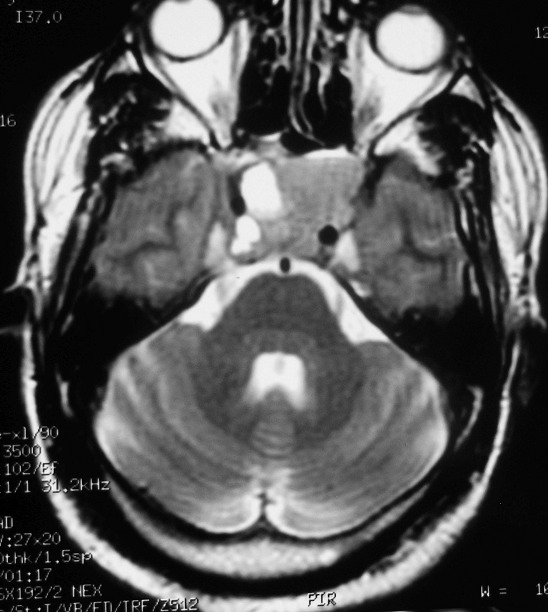
Coupe axiale pondérée en T2 montrant l'hypersignal T2 intermédiaire déclive de la lésion kystique; la tige pituitaire est refoulée vers le haut et l'avant

**Figure 3:**
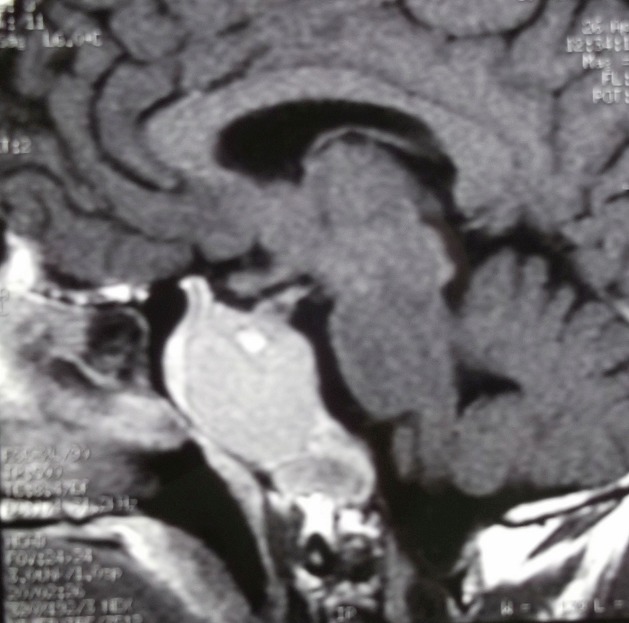
Coupe sagittale pondérée en T1 après injection de gadolinium: on ne note pas de rehaussement de la portion solide ou des septas intra lésionnels

Il s'agit d'un patient de 44 ans sans antécédent pathologique particulier qui s'est présenté aux urgences pour un syndrome d'hypertension intracrânienne fait de céphalées chroniques et une baisse progressive de l'acuité visuelle. L'examen ophtalmologique retrouve un 'dème papillaire stade 2 bilatéral avec une mesure de l'acuité visuelle estimée à 6/10 à droite et à 5/10 à gauche. Le reste de l'examen somatique est sans particularités. Au bilan biologique, on note une perturbation du bilan hormonal à type d'hyperprolactinémie à 200ng/ml sans autre anomalie des autres hormones antéhypophysaires notamment ACTH et GH. Une IRM cérébrale a été réalisée; elle met en évidence un volumineux processus lésionnel kystique centré sur le corps et la base du sphénoïde avec importante extension intra sellaire. Cette lésion est faite de multiples logettes kystiques en hypersignal T1spontané (Figure 1) avec niveaux dont la partie déclive est en hypersignal T2 (Figure 2) refoulant la tige pituitaire vers le haut et l'avant. On ne note pas de rehaussement des parois kystiques (Figure 3). Cet aspect radiologique est typique du kyste osseux anévrysmal dans une localisation atypique qui est le clivus et le corps du sphénoïde. Cependant et dans un premier temps, cette lésion a été étiquetée comme un macro adénome en transformation nécrotico-hémorragique. Le patient a été mis sous agonistes dopaminergiques et devant la non amélioration de la symptomatologie et l'absence de fonte tumorale attendue, une relecture du dossier a été faite ayant conclu définitivement à un kyste osseux anévrysmal. Ce diagnostic a été retenu sur un faisceau d'arguments clinico radiologiques en pré opératoire avec confirmation histologique de la pièce opératoire. L'hyperprolactinémie retrouvée chez le patient est liée à un « phénomène d'entrainement » du fait de la compression de la lésion sur la tige pituitaire.

## Discussion

Le kyste osseux anévrysmal (KOA) est une tumeur relativement bien différenciée du sujet jeune. Les sièges les plus fréquents se situent au niveau du rachis, du fémur et du tibia. Ils sont habituellement localisés au niveau des métaphyses des os longs. A l'instar de notre observation, dans seulement 3% des cas, le KOA se localise au niveau de la base du crane et notamment le basisphénoide [3]. Sur le plan physiopathologique, il s'agit d'un processus hyperplasique, secondaire à une hémorragie sous-périostée ou intra-osseuse, qui stimule la prolifération d'un tissu réactionnel avec activation ostéoclastique. Ce processus peut se pérenniser aboutissant ainsi à une lésion rapidement invasive ou alors disparaître progressivement avec à long terme une ossification des parois de la cavité [3]. Sur le plan clinique, au niveau du sphénoïde, le kyste osseux anévrysmal est insidieux et peu bruyant. La symptomatologie clinique est liée à la destruction osseuse environnante et aux structures anatomiques comprimées au contact de la lésion en première ligne desquelles on trouve par ordre de fréquence les céphalées, une paralysie des paires crâniennes ou des signes d'obstruction nasale [1, 3, 4]. Dans de rares cas, des perturbations du bilan hormonal peuvent être retrouvées ce qui était le cas dans notre observation; elles seraient due à un effet d'entrainement par compression de la tige pituitaire. L'aspect en imagerie du kyste osseux anévrysmal est quasi similaire quelque soit sa localisation. Il se présente sur la radiographie standard comme une lésion expansive faite de multiple logettes kystiques et hémorragiques avec niveau liquide-liquide, soufflant l'os, responsable d'un amincissement de la corticale osseuse sans véritable réaction périostée à son contact [1, 4].

En intracrânien cependant, il est impossible de se suffire d'une radiographie standard seule et le recours à l'imagerie en coupe notamment le scanner et l'imagerie résonance magnétique (IRM) est obligatoire et quasi systématique d'une part pour le diagnostic positif mais surtout pour le diagnostic différentiel avec les autres lésions de l'étage moyen de la base du crane. Le scanner met en évidence une lésion hétérogène avec multiples logettes liquidiennes, non rehaussées après injection de produit de contraste iodé et responsable d'une lyse du sphénoïde [1, 4]. L'IRM met également en évidence ces logettes kystiques avec niveau, des septas intra lésionnels, un signal hétérogène sur les séquences T1 et T2 et un rehaussement faible voire nul sur les séquences T1 après injection de gadolinium. Les niveaux liquides traduisent l'hémorragie intra lésionnelle et le saignement chronique d'âges différents réalisant des sédiments et sont donc mieux appréciés en IRM. Ainsi, elles se manifestent par un signal élevé en T1 et hétérogène en T2 [4]. Le diagnostic différentiel en imagerie se fait avec le chordome dans son siège clival et moins fréquemment avec le macroadénome invasif en transformation hémorragique. En effet, ce dernier se présente comme une lésion intra et suprasellaire avec une seule logette kystique siège d'un niveau avec sédiment et on retrouvera une portion charnue qui se rehausse modérément par rapport à la glande hypophysaire saine. Le chordome quant à lui se présente comme une lésion centrée sur le clivus lytique en hyposignal T1, hypersignal hétérogène T2, rehaussée de façon intense et hétérogène sans logette kystique en son sein. Le traitement du KOA consiste en une résection chirurgicale complète par voie transcrânienne ou transphénoidale [1, 4]. C'est l'examen histologique de la pièce d'exérèse qui tranche en retrouvant une masse hémorragique spongieuse faite d'épais septas gris rougeâtres parfois blancs qui délimitent des lacunes remplies de sang [5]. Il a été décrit des rares cas de résorption spontanée du KO, suite à des agressions mineures (biopsie), à une irradiation, après embolisation, ou à l'injection de substances ostéo-inductrices.

## Conclusion

Le KOA du sphénoïde n'est pas une lésion fréquente de la base du crane. Cependant, il faut savoir l'évoquer sur l'IRM devant une lésion expansive multi loculée avec de multiples niveaux liquide-liquide. Ainsi, l'IRM joue définitivement un rôle important dans le diagnostic positif du KOA mais également dans le diagnostic différentiel en permettant d'écarter aisément les autres lésions du sphénoïde.

## Conflits d’intérêts

Les auteurs ne déclarent aucun conflit d'intérêts.
